# Positive Youth Development in Adolescence

**DOI:** 10.34763/jmotherandchild.20212503SI.edit.2021_25_03SI_3

**Published:** 2022-03-01

**Authors:** Teresa Santos

**Affiliations:** 1 Clinical and Health Psychology Universidade Europeia, Lisbon CatolicaMed Platform/Centre for Interdisciplinary Research in Health of Universidade Católica Portuguesa (UCP), Lisbon Portugal

The *Journal of Mother and Child*’s special issue has been focused on the current direction of positive development, resilience and well-being in adolescence. Therefore, it is important to take a view on the current papers taken up by the authors that better help to understand these topics. In addition to highlighting the relevance of research concerning positive development, resilience and well-being, this commentary points out that an integral and gender-sensitive healthy perspective for youths, based on interdisciplinary and transdisciplinary work that relies on supportive contexts to more likely achieve positive outcomes, is needed for the future [1].

Adolescence is a critical moment to establish health behaviours and healthy paths that might have a positive impact in later adulthood well-being [2].

In the past century, the studies on youths were mostly based on a “deficit perspective” and risk behaviours, which had a major influence on policies, research and practice. Accordingly, the positive development concept was mainly defined by the absence or the decrease of problems. In the last two decades, this perspective improved and a limited impact was acknowledged, if youth programs and interventions were mostly focused on risks and vulnerabilities [3]. The idea to prevent problems but also to promote a healthy youth development led to strength-based models, stressing the effective empowerment in diverse contexts [4, 5], as for example the Positive Youth Development (PYD). PYD emphasizes the importance of strengthening internal and external developmental assets contained in the social ecology of youth’s networks and opportunities [6, 7]. Such theoretical perspectives have presented positive indicators such as the Model of the Search Institute’s Developmental Assets (40, comprised on external and internal assets) [4] and the Five Cs model of PYD (Competence, Confidence, Character, Connection and Caring) [5].

Thus, contemporary models of youth development and problem prevention can be generally grouped into one of three types of approaches: prevention, resiliency and positive youth development [6]. In 2010, an integrative model of pathways toward healthy development (combining resilience and PYD) was also developed [8]. This model included a protective path (drawn from the resilience research and comprising risk and protection) and a promoting path (drawn from the PYD research and including assets). Both pathways lead to a broad category of healthy development. Another framework named the Behaviour Change Wheel Model (BCW) additionally suggested that for behavioral change to happen at least three components were needed: capacity, motivation and opportunity (connected with the context). This model reinforced the context as a key factor for the design and implementation of effective interventions [9]. In sum, effective youth programs must include the “Big Three” constituents, namely youth participation (opportunities and leadership); skills building (emphasis on the development of life skills); and adult mentorship (a context of sustained and caring adult-youth relationships) [5].

Self-regulation has been positively associated with well-being, positive youth development and positive outcomes in adulthood [10]. Self-regulation is one important protective factor for positive development along with resilience, and both variables have a significant and positive connection. Yet there is no single factor, isolated program or strategy that promotes resilience and provides all opportunities for a successful youth development [11, 5].

In this issue, there are papers focusing on the role of family for youths: Dimitrova & Kotzeva point out the relevant role of family-related characteristics for risk behaviours, and Pianarosa & Davidson emphasize that family support, self-reported health and mental health were common factors strongly associated with reporting a happy home life. Other authors Gajda, Berkowska & Małkowska-Szkutnik draw attention to the role of the context for youths, namely the reality in Hospital Schools and more specifically during the COVID-19 lockdown.

The research allowed increasing the understanding on relevant strategies for youth development, namely to set realistic goals and to learn from mistakes; to increase self-control strategies (thoughts, emotions, impulses and behaviour); to work on youth’s strengths; to decrease anxiety; and to encourage positive adaptation. All of these can help to cope positively with adverse situations and also to build an optimistic life plan, facilitating to attain a happy and healthy life. Finally, it is crucial to keep in mind that youths are the most important assets in the world. Thus, for the future it is particularly beneficial to continue to work on the identification of pertinent variables that promote positive youth development because, when a positive development occurs, youths can power to benefit themselves, families, communities and societies, and the consequent effects can last for generations. To invest in youths can signify a highly cost-effective opportunity towards positive changes [12].
